# Evaluating the Nolla Method for Dental Age Estimation in Children from Northwestern Romania

**DOI:** 10.3390/children12010069

**Published:** 2025-01-07

**Authors:** Ligia Ioana Moga, Abel Emanuel Moca, Raluca Iurcov, Dan Slăvescu, Ligia Luminița Vaida

**Affiliations:** 1 Doctoral School of Biomedical Sciences, University of Oradea, 1 Universității Street, 410087 Oradea, Romania; ligia_lazar2004@yahoo.com; 2 Department of Dentistry, Faculty of Medicine and Pharmacy, University of Oradea, 10 Piața 1 Decembrie Street, 410073 Oradea, Romania; riurcov@uoradea.ro (R.I.); slavescudan@uoradea.ro (D.S.); ligia_vaida@uoradea.ro (L.L.V.)

**Keywords:** dental age, Nolla method, Romania

## Abstract

Background/Objectives: Dental age estimation plays a critical role in pediatric dentistry, orthodontics, and forensic medicine. The Nolla method, widely applied globally, has shown variable accuracy across different populations. This study aimed to evaluate the applicability and accuracy of the Nolla method in estimating the dental age of Romanian children and to identify potential discrepancies between dental and chronological ages. Methods: A retrospective analysis was conducted on 860 panoramic radiographs from pediatric patients aged 3–15.9 years in Oradea, Romania. The Nolla method was applied to estimate dental age, and the results were compared with chronological age. Statistical analyses, including Wilcoxon signed-rank and Mann–Whitney U tests, were performed to evaluate the accuracy and consistency of the Nolla method. Results: The study analyzed 860 panoramic radiographs (356 boys and 504 girls). The average chronological age was 9.95 ± 2.48 years, while the average dental age, as estimated using the Nolla method, was 8.43 ± 2.13 years. Dental age was consistently lower than chronological age, with a median difference of 1.5 years (IQR: 0.9–2.2 years). Among the 13 age groups, the highest representation was found in the 8–8.9-year (14.7%) and 9–9.9-year (13.3%) groups. Gender differences were statistically significant (*p* < 0.001); girls demonstrated a larger median discrepancy of 1.7 years (IQR: 1.1–2.3 years) compared to boys at 1.15 years (IQR: 0.6–1.8 years). Notably, discrepancies increased with age, peaking at 2.6 years in the 14–14.9-year group (4.7% of the sample). The youngest group (3–3.9 years) showed the smallest difference of 0.3 years. Significant differences between chronological and dental ages were observed in 87.5% of the sample. Conclusions: The Nolla method consistently underestimated dental age in Romanian children, with greater discrepancies in older age groups and among girls. These findings highlight the need for the population-specific calibration of the method to improve its accuracy in both clinical and forensic contexts.

## 1. Introduction

Determining age is a crucial step in gathering information for various dental procedures, including pediatric dentistry and orthodontics, as well as in medical fields such as endocrinology, disease diagnosis, and forensic medicine [1]. Traditionally, chronological age has been assessed using biometric tests, comparing results to standard curves derived from large cohorts of healthy individuals. However, skeletal and dental age are now recognized as more reliable indicators [2].

The radiological appearance of cervical vertebrae provides a useful measure of bone development. Over the years, methods for evaluating cervical vertebrae have evolved [3,4], culminating in the cervical vertebral maturation (CVM) method. This approach evaluates the second, third, and fourth cervical vertebrae using lateral cephalometric radiographs [5]. These radiographs are indispensable for orthodontic diagnosis and treatment planning, allowing for the assessment of skeletal maturity without additional radiation exposure [6]. Nonetheless, further research is needed to clarify the associations between age and CVM developmental stages [7,8].

Studies indicate that chronological age correlates more strongly with tooth development than with body size or skeletal maturation, likely because skeletal age is more strongly influenced by environmental factors. Dental development, characterized by the timing and sequence of tooth eruption, is affected by factors such as caries, premature loss or the prolonged retention of primary teeth, misalignment, and dental ankylosis. Tooth development progresses in a gradual and cumulative manner. Numerous methods have been proposed for the radiographic analysis of dental age, with the Nolla and Demirjian methods gaining widespread popularity over recent decades [2].

These methods involve identifying stages of mineralization on radiographs and comparing them to established standards to estimate age [9]. Various radiographic techniques are employed for age estimation, including intraoral periapical, lateral oblique, cephalometric, panoramic radiographs, digital imaging, and advanced imaging technologies [9]. To ensure accuracy, the radiographs must adequately capture the teeth under evaluation, encompassing all relevant stages of development [9,10].

The Demirjian method, introduced in 1973 following a study of 2928 French Canadian children, classifies dental development into eight stages (A–H) [11]. Alternatively, the Nolla method, developed in 1952 based on a study of 3402 children from Michigan, categorizes tooth development into 10 stages (1–10) using panoramic radiographs [12,13]. The Nolla method has been widely applied and adapted across different populations [12,14,15,16]. Dental age, as estimated by these methods, is often compared to chronological age to assess their validity in specific populations [17].

While the Nolla method has been applied globally, variations in results highlight the need for population-specific validation. In Romania, an accurate method for dental age estimation is particularly necessary. Previous studies have applied the Demirjian method, but it tended to overestimate dental age in Romanian populations [8]. Therefore, it remains essential to evaluate and validate alternative methods for accuracy in this demographic.

The primary aim of this study was to evaluate the accuracy and applicability of the Nolla method for dental age estimation in a sample of Romanian children from northwestern Romania. Specifically, the study sought to achieve the following:

- Compare the dental age estimated using the Nolla method with the chronological age of the participants to assess the method’s precision.
- Determine whether the Nolla method provides consistent and reliable results in the Romanian pediatric population, given potential genetic, environmental, and socio-economic influences on dental development.
- Highlight any discrepancies or patterns that may necessitate adaptations or recalibrations of the Nolla method for this specific population.

## 2. Materials and Methods

### 2.1. Ethical Considerations

The study adhered to the ethical principles of the 1964 Declaration of Helsinki and its subsequent amendments. Approval was obtained from the Research Ethics Committee of the Faculty of Medicine and Pharmacy at the University of Oradea (approval no. CEFMF/1, dated 30 July 2024).

### 2.2. Sample Selection

This retrospective study analyzed digital panoramic radiographs obtained from a pediatric patient sample in Bihor, northwestern Romania, between 1 August 2024 and 30 September 2024. The radiographs were sourced from a single dental practice in Oradea and were originally taken for diagnostic purposes related to pedodontic or orthodontic treatment, not specifically for this study. Radiographs were captured using the Soredex Cranex Novus Panorex (Soredex, Milwaukee, WI, USA) system. Following an initial clinical examination, panoramic radiographs were recommended for patients requiring further treatment.

Informed consent was obtained from parents or caregivers, permitting the use of radiographs from minor patients for research purposes. Each radiograph was accompanied by the patient’s name, date of birth, and the date of the radiographic examination. Additionally, as part of the retrospective nature of this study, researchers had access to the patients’ full medical records, which provided supplementary information that aided in meeting the inclusion and exclusion criteria of the study. Digital panoramic radiographs were stored in JPEG (Joint Photographic Experts Group) format.

A total of 945 panoramic radiographs were initially reviewed. Panoramic radiographs of patients aged 3–15.9 years (with documented dates of birth and radiographic evaluations), taken for the diagnosis and treatment of dental issues, were included.

Patients were excluded if they were non-cooperative, had documentation of birth dates or radiographic evaluation dates, or possessed syndromes or systemic conditions affecting tooth eruption and development.

Based on these criteria, 85 radiographs were excluded, leaving a final sample of 860 radiographs. The remaining patients were divided into 13 age groups, each spanning one year (e.g., 3.0–3.9 years and 4.0–4.9 years). This grouping enabled a more precise comparison of chronological and dental ages and any discrepancies between them.

To determine the minimum necessary sample size for a population of children aged 3 to 15 in Bihor County, Romania, which is approximately 92,000 [18], we conducted a sample size calculation. This calculation is crucial for ensuring that the study results are statistically significant within specified parameters.

For the calculation, we set the following parameters: a 95% confidence level, which corresponds to a Z-score of 1.96 and is commonly used in social science research; a margin of error at 5% (±5%), a standard choice in various research scenarios; and a proportion (*p*) of 0.5, representing the worst-case scenario that maximizes the sample size requirement, typically used when the actual proportion is unknown.

The formula used to calculate the initial sample size for an infinite population was the following:

$$n=\frac{Z^{2}\cdot p\cdot (1-p)}{E^{2}}$$

where *n* is the sample size, *Z* is the Z-score (1.96 for 95% confidence), *p* is the estimated proportion of the attribute present in the population, and *E* is the margin of error.

For finite populations, this initial sample size needs to be adjusted using the following formula:

$$n_{adjusted}=\frac{n}{1+\frac{n-1}{N}}$$

where *N* is the total population size.

Using the above formulas, the initial sample size calculated was approximately 384. Considering the finite population of 92,000 children, the adjusted sample size was approximately 383. This adjusted size reflects the reduction in variability expected when the population from which the sample is drawn is not infinitely large.

### 2.3. Evaluation of Chronological and Dental Ages

Chronological age was calculated as the difference between the patient’s date of birth and the date of the radiographic examination.

**Figure 1:**
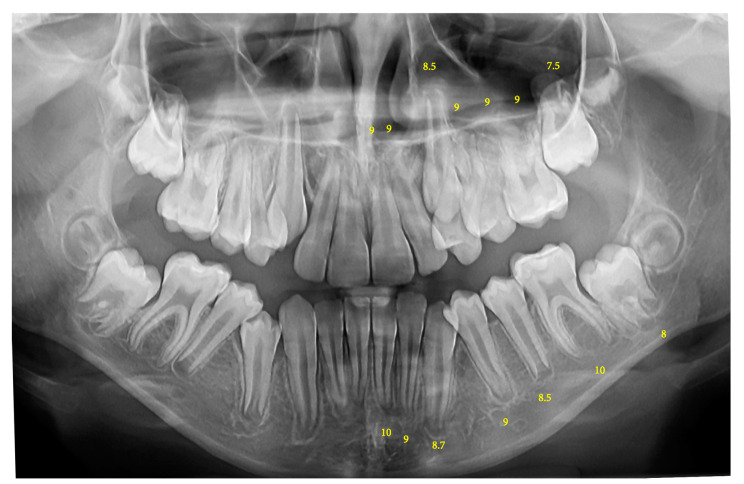
Scoring teeth on a panoramic radiograph using the Nolla method.

Dental age was estimated using the Nolla method, which evaluates the development of the seven permanent teeth on the left side of the maxilla and mandible, excluding the third molar. The Nolla method assigns developmental stages numbered 1 to 10, with stage 1 indicating the presence of a crypt and stage 10 indicating the completion of root development with apical closure. An additional stage 0, indicating the absence of a crypt, was also noted when applicable [13] (Figure 1).

The developmental stages of the teeth were summed to calculate a total dental maturation score, which was then matched to corresponding dental age norms based on gender-specific tables developed by Nolla. To ensure consistency and eliminate inter-operator variability, a single investigator performed all radiographic analyses and assessments of dental age.

### 2.4. Statistical Analysis

Statistical analyses were conducted using IBM SPSS Statistics 25 and Microsoft Office Excel/Word 2021. The distribution of quantitative variables was assessed with the Shapiro–Wilk test. Depending on the distribution, these variables were expressed as either means with standard deviations or medians with interquartile ranges. Categorical variables were presented as absolute values or percentages.

For independent quantitative variables, the Mann–Whitney U test or Kruskal–Wallis H test was applied, as the data did not follow a parametric distribution. Correlations between variables were evaluated using Spearman’s Rho correlation coefficient. When significant results were identified in independent variable testing, Dunn–Bonferroni tests were employed as post hoc analyses. To compare chronological and dental ages, the Wilcoxon signed-rank test was used to identify significant differences.

## 3. Results

This study analyzed a total of 860 panoramic radiographs, comprising 356 from boys and 504 from girls. The average chronological age of the sample was 9.95 ± 2.48 years, with a median of 9.9 years and a range of 3 to 15 years. The average dental age, as estimated using the Nolla method, was 8.43 ± 2.13 years, with a median of 8 years and a range of 2 to 16 years.

Table 1 presents the distribution of patients across age groups. The majority of patients were concentrated in the 8–8.9-year (14.7%), 9–9.9-year (13.3%), and 11–11.9-year (13.3%) age groups. Among girls, the highest number of patients was observed in the 9–9.9-year age group, with 74 patients (8.6%). For boys, the largest group was in the 8–8.9-year range, comprising 66 patients (7.7%). The smallest number of patients for both boys and girls was recorded in the youngest age group (3–3.9 years) and the oldest age group (15–15.9 years).

Table 2 presents the average chronological and dental ages for each age group, along with the progression of these values across the sample. The average chronological age ranged from 3.30 years in the youngest age group (3–3.9 years) to 15.00 years in the oldest (15–15.9 years), with standard deviations varying between 0.26 years in the youngest group and 0 years in the oldest. Similarly, the average dental age ranged from 3 years in the youngest age group to 16 years in the oldest. The standard deviation for dental age ranged from 0 in both the youngest and oldest age groups to a maximum of 1.07 years in the 14–14.9-year age group. This table consolidates key data on the progression and variability of chronological and dental ages across the sample population.

Table 3 demonstrates that the distributions of chronological age and dental age were non-parametric, as indicated by the Shapiro–Wilk test (*p* < 0.05). The Wilcoxon signed-rank test revealed significant differences between the two measures (*p* < 0.001), with dental age values being consistently lower than chronological age values. The median difference was 1.5 years, with an interpercentile range of 0.9–2.2 years.

Table 4 presents the differences between chronological age and dental age across the age groups. The Shapiro–Wilk test indicated that the distribution of differences was non-parametric in most age groups (*p* < 0.05). Analysis using the Kruskal–Wallis H test revealed significant differences between groups (*p* < 0.001). The distribution observed in the box plot, along with post hoc test results, showed that the differences between chronological age and dental age remained relatively constant and small in patients aged 3–7.9 years, with no significant variation among these age groups. However, beginning at the 8–8.9-year age group, the differences increased, peaking at 11–11.9 years. Elevated differences persisted until the 14–14.9-year age group.

The Shapiro–Wilk test confirmed that the distribution of differences was non-parametric in both gender groups (*p* < 0.05). The Mann–Whitney U test revealed significant differences (*p* < 0.001), with girls showing larger discrepancies (median = 1.7 years; interpercentile range = 1.1–2.3 years) compared to boys (median = 1.15 years; interpercentile range = 0.6–1.8 years). These findings are summarized in Table 5.

## 4. Discussion

This study evaluated the accuracy and applicability of the Nolla method for dental age (DA) estimation in a sample of Romanian children from Oradea. The findings revealed significant discrepancies between chronological age (CA) and DA, with DA values consistently lower across most age groups. The median difference was 1.5 years, underscoring the need for the population-specific validation and adaptation of age estimation methods. Population-specific validation involves applying a method to a new population different from the original, with the goal of refining DA estimation to better predict CA [19]. While the Demirjian method has been previously validated on a larger Romanian sample [8], it was found to overestimate CA, necessitating the exploration of alternative methods like Nolla.

In this study, patients ranged in age from 3 to 15.9 years, with the upper age limit chosen because it marks the end of the pubertal growth spurt in most children [20]. The lower age limit of 3 years was set because no patients younger than 3 years old, who had undergone panoramic radiographs, were identified in the dental offices from which the radiographs were sourced. Regarding this lower age limit, there is no standardized minimum age recommended for performing panoramic radiographs; rather, the decision should be made based on several factors [21,22]. The vast majority of patients were between 7 and 13 years old, as these are the ages at which panoramic radiographs are commonly performed [23]. Although the sample sizes for the age groups of 3–3.9 years, 4–4.9 years, 5–5.9 years, and 15–15.9 years were relatively small, which could limit the statistical interpretation of the data, this reflects the actual distribution of patients presenting for radiographs in clinical practice. The authors recognize the need for further research with larger samples from these age groups to verify and expand upon our findings. This study aims to provide a basis for such future investigations.

In this study, DA was consistently lower than CA across most age groups, a trend that aligns with previous research. For instance, Paz Cortés et al. (2019) reported DA underestimations using the Nolla method in a Spanish cohort aged 4 to 14 years [16], while Duruk et al. (2022) and Miloglu et al. (2011) observed similar findings in Eastern Turkish children [14,24]. In contrast, studies from northern and northeastern China identified overestimations of DA with the Nolla method [25,26]. Globally, findings are mixed, with some studies reporting underestimations similar to those observed in this research [27]. These variations highlight the influence of genetic, environmental, and socio-economic factors on dental development [28,29].

Sexual dimorphism in dental development was evident in this study, with girls showing a larger median discrepancy between CA and DA (1.7 years) compared to boys (1.15 years). This aligns with previous findings suggesting earlier dental maturation in girls, as reflected in Tanner Stage 3, a critical milestone in pubertal development [30,31,32,33]. These results underscore the importance of gender-specific considerations when applying the Nolla method [34].

The use of panoramic radiographs in this study mirrors the standard approach in most Nolla-based DA estimation studies [14,16,24]. However, advancements in imaging, such as the application of 3D cone-beam computed tomography (CBCT), have demonstrated the potential for enhanced accuracy. Zirk et al. (2021) found that CBCT provided more detailed staging data compared to traditional 2D panoramic radiographs, highlighting the potential for 3D imaging to improve DA estimation in forensic and clinical contexts [35].

Clinically, these findings have implications for pediatric dentistry, orthodontics, and forensic age estimation in Romania. The underestimation of DA using the Nolla method may affect treatment planning, particularly for orthodontic interventions where precise developmental staging is essential [36]. In order to address the crucial role of dental and skeletal development assessments in orthodontic treatment planning, it is important to emphasize that these evaluations are paramount irrespective of their divergence from chronological age. The accurate determination of dental and skeletal maturity allows for the tailoring of interventions that are optimally timed to the physiological growth stages of individual patients, enhancing treatment effectiveness and efficiency. This study’s findings, which identify discrepancies between DA and CA, underscore the necessity of integrating development-specific data into clinical practices. Such integration aids in aligning treatment plans with the patient’s unique developmental timeline, thereby optimizing therapeutic outcomes [37]. This approach is supported by a wide body of research that confirms the variability of growth patterns among individuals and highlights the limitations of relying solely on chronological age for orthodontic decision making [38,39]. The nuanced understanding of growth and development not only facilitates more precise treatment timing but also contributes to the broader application of orthodontic practices tailored to individual growth dynamics. Consequently, the findings of this study advocate for the adaptation and calibration of age estimation methods, like the Nolla method, to incorporate region-specific developmental characteristics, thus enhancing the accuracy and relevance of such tools in both clinical and forensic applications [19,40].

In forensic contexts, the calibration of the Nolla method to account for Romanian-specific characteristics is crucial, as DA estimation remains a vital tool for rapid and reliable forensic identification [41]. The use of a calibrated method for determining dental age can be valuable in forensic medicine for identifying bodies in mass disasters and human remains in both archaeological and criminal investigations [42,43,44]. Additionally, it can be useful in cases of child trafficking or for addressing issues related to immigration and refugees [45,46].

This study’s strengths include a robust sample size and a focus on a specific population, offering valuable insights into the applicability of the Nolla method in Romania. However, limitations such as its retrospective design, reliance on a single dental practice, and the use of a single method for DA estimation may restrict generalizability. Future research should validate these findings in larger, more diverse Romanian cohorts and explore integrating multiple methods to improve accuracy. Another limitation of this study is the dependence on the clinician’s expertise and ability to accurately assign scores. As with all clinical scoring systems, the results of our study could be influenced by the subjective judgment and skill level of the clinicians involved. This introduces potential variability in the scoring process, which could affect the consistency and reliability of the findings. Longitudinal studies investigating genetic and environmental influences on dental development in Romanian children could provide further advancements in this field.

## 5. Conclusions

This study demonstrated that the Nolla method underestimated the dental age of Romanian children across the analyzed age groups, with varying degrees of underestimation. The greatest discrepancy was observed in the 14–14.9-year age group, while the smallest difference occurred in the youngest group (3–3.9 years). Both girls and boys experienced underestimation of dental age, with girls showing larger discrepancies than boys.

These findings suggest that while the Nolla method remains a widely used tool for dental age estimation, its applicability in the Romanian pediatric population may require recalibration. Adapting the method to better align with the specific developmental characteristics of this population or exploring alternative dental age assessment methods could enhance accuracy and reliability in both clinical and forensic applications.

## Figures and Tables

**Table 1:** Distribution of patients based on age group.

Age Group	Girls (n, %)	Boys (n, %)	Total	Percentage
3–3.9 years	2 (0.2%)	4 (0.5%)	6	0.7%
4–4.9 years	4 (0.5%)	8 (0.9%)	12	1.4%
5–5.9 years	12 (1.4%)	10 (1.2%)	22	2.6%
6–6.9 years	36 (4.2%)	24 (2.8%)	60	7%
7–7.9 years	50 (5.8%)	42 (4.9%)	92	10.7%
8–8.9 years	60 (7%)	66 (7.7%)	126	14.7%
9–9.9 years	74 (8.6%)	40 (4.7%)	114	13.3%
10–10.9 years	70 (8.1%)	34 (4%)	104	12.1%
11–11.9 years	66 (7.7%)	48 (5.6%)	114	13.3%
12–12.9 years	66 (7.7%)	32 (3.7%)	98	11.4%
13–13.9 years	40 (4.7%)	30 (3.5%)	70	8.1%
14–14.9 years	22 (2.6%)	18 (2.1%)	40	4.7%
15–15.9 years	2 (0.2%)	0 (0%)	2	0.2%

**Table 2:** The average CA and DA based on age group.

Age Group	CA (In Years)	SD (In Years)	DA (In Years)	SD (In Years)
3–3.9 years	3.30	0.26	3.00	0.00
4–4.9 years	4.48	0.32	3.50	0.79
5–5.9 years	5.39	0.31	4.55	0.51
6–6.9 years	6.46	0.31	6.13	0.77
7–7.9 years	7.51	0.30	6.54	0.58
8–8.9 years	8.42	0.26	7.21	0.69
9–9.9 years	9.46	0.31	7.98	0.78
10–10.9 years	10.37	0.26	8.71	0.79
11–11.9 years	11.43	0.29	9.54	1.01
12–12.9 years	12.41	0.30	10.2	0.90
13–13.9 years	13.37	0.30	11.43	0.87
14–14.9 years	14.45	0.29	11.85	1.07
15–15.9 years	15.00	0.00	16	0

CA—chronological age; DA—dental age; SD—standard deviation.

**Table 3:** Comparison of CA versus DA.

Parameter	Mean Age ± SD	Median (IQR)	*p* *
Chronological age (*p* < 0.001 **)	9.94 ± 2.48	9.9 (8.11–11.8)	<0.001
Dental age (*p* < 0.001 **)	8.43 ± 2.13	8 (7–10)	
Average difference	1.52 ± 1.01	1.5 (0.9–2.2)	-

CA—chronological age; DA—dental age; SD—standard deviation; * related-sample Wilcoxon signed-rank Test; ** Shapiro–Wilk test.

**Table 4:** Differences between CA and DA based on age group.

Age	Mean ± SD	Median (IQR)	Mean Range	*p* *
3–3.9 years (*p* = 0.167 **)	0.3 ± 0.26	0.3 (0–0.6)	100.83	<0.001
4–4.9 years (*p* = 0.011 **)	0.98 ± 0.68	0.9 (0.4–1.1)	272.33	
5–5.9 years (*p* = 0.001 **)	0.84 ± 0.72	0.9 (0.1–1.7)	258.77	
6–6.9 years (*p* = 0.060 **)	0.32 ± 0.82	0.2 (−0.1–0.9)	154.70	
7–7.9 years (*p* < 0.001 **)	0.97 ± 0.68	1 (0.4–1.6)	291.91	
8–8.9 years (*p* = 0.007 **)	1.21 ± 0.71	1.3 (0.7–1.7)	356.40	
9–9.9 years (*p* = 0.035 **)	1.48 ± 0.76	1.6 (0.9–2.12)	428.34	
10–10.9 years (*p* = 0.001 **)	1.66 ± 0.78	1.6 (1.13–2.1)	474.06	
11–11.9 years (*p* = 0.001 **)	1.89 ± 1.02	2.1 (1.07–2.6)	524.61	
12–12.9 years (*p* = 0.001 **)	2.21 ± 0.94	2.1 (1.4–3)	588.48	
13–13.9 ani (*p* = 0.093 **)	1.95 ± 0.93	2.11 (1.11–2.5)	538.24	
14–14.9 ani (*p* = 0.009 **)	2.60 ± 1.07	3 (1.62–3.35)	658.15	
15–15.9 ani (*p* = - **)	−1 ± 0	−1 (−1–−1)	3.50	

CA—chronological age; DA—dental age; SD—standard deviation; IQR—interquartile range; * Kruskal–Wallis H test; ** Shapiro–Wilk test.

**Table 5:** Differences between CA and DA based on gender.

Gender	Mean Age ± SD	Median (IQR)	*p* *
Girls (*p* = 0.011 **)	1.707 ± 1.009	1.7 (1.1–2.3)	<0.001
Boys (*p* = 0.001 **)	1.256 ± 0.965	1.15 (0.6–1.8)	

SD—standard deviation; * Mann–Whitney U test, ** Shapiro–Wilk test, SD—standard deviation, IQR—interquartile range.

## Data Availability

The data presented in this study are available on request from the corresponding authors. The data are not publicly available due to privacy reasons.
